# Do We Need to Use Bats as Bioindicators?

**DOI:** 10.3390/biology10080693

**Published:** 2021-07-21

**Authors:** Danilo Russo, Valeria B. Salinas-Ramos, Luca Cistrone, Sonia Smeraldo, Luciano Bosso, Leonardo Ancillotto

**Affiliations:** Wildlife Research Unit, Dipartimento di Agraria, Università degli Studi di Napoli Federico II, Via Università, 100, 80055 Portici, Italy; valeria.salinasramos@unina.it (V.B.S.-R.); luca.cistrone@gmail.com (L.C.); sonia.smeraldo@unina.it (S.S.); leonardo.ancillotto@unina.it (L.A.)

**Keywords:** biodiversity, Chiroptera, climate change, environment, foraging, forest, habitat, river, urban

## Abstract

**Simple Summary:**

Bioindicators are organisms that react to the quality or characteristics of the environment and their changes. They are vitally important to track environmental alterations and take action to mitigate them. As choosing the right bioindicators has important policy implications, it is crucial to select them to tackle clear goals rather than selling specific organisms as bioindicators for other reasons, such as for improving their public profile and encourage species conservation. Bats are a species-rich mammal group that provide key services such as pest suppression, pollination of plants of economic importance or seed dispersal. Bats show clear reactions to environmental alterations and as such have been proposed as potentially useful bioindicators. Based on the relatively limited number of studies available, bats are likely excellent indicators in habitats such as rivers, forests, and urban sites. However, more testing across broad geographic areas is needed, and establishing research networks is fundamental to reach this goal. Some limitations to using bats as bioindicators exist, such as difficulties in separating cryptic species and identifying bats in flight from their calls. It is often also problematic to establish the environmental factors that influence the distribution and behaviour of bats.

**Abstract:**

Bats show responses to anthropogenic stressors linked to changes in other ecosystem components such as insects, and as K-selected mammals, exhibit fast population declines. This speciose, widespread mammal group shows an impressive trophic diversity and provides key ecosystem services. For these and other reasons, bats might act as suitable bioindicators in many environmental contexts. However, few studies have explicitly tested this potential, and in some cases, stating that bats are useful bioindicators more closely resembles a slogan to support conservation than a well-grounded piece of scientific evidence. Here, we review the available information and highlight the limitations that arise in using bats as bioindicators. Based on the limited number of studies available, the use of bats as bioindicators is highly promising and warrants further investigation in specific contexts such as river quality, urbanisation, farming practices, forestry, bioaccumulation, and climate change. Whether bats may also serve as surrogate taxa remains a controversial yet highly interesting matter. Some limitations to using bats as bioindicators include taxonomical issues, sampling problems, difficulties in associating responses with specific stressors, and geographically biased or delayed responses. Overall, we urge the scientific community to test bat responses to specific stressors in selected ecosystem types and develop research networks to explore the geographic consistency of such responses. The high cost of sampling equipment (ultrasound detectors) is being greatly reduced by technological advances, and the legal obligation to monitor bat populations already existing in many countries such as those in the EU offers an important opportunity to accomplish two objectives (conservation and bioindication) with one action.

## 1. Introduction

The scientific literature is crowded with definitions for “bioindicator”. According to the Oxford Dictionary of Zoology, an indicator species is “a species of narrow amplitude with respect to one or more environmental factors and that is, when present, indicative of a particular environmental condition or set of conditions” [1]. An even more general definition, which we adopt for the goals of this paper, might include processes beyond organisms and see bioindicators as “any biological entity (taxon or process) that responds in some way to environmental characteristics or their alteration”.

Many bioindicator subcategories have been proposed according to the information conveyed by such responses. For instance, [2] distinguished between environmental, ecological, and biodiversity indicators. While ecological bioindicators are used to detect and monitor the effects of a stressor on the biota, environmental indicators allow detecting and monitoring changes in a given environmental state, and biodiversity indicators make it possible to identify and monitor species diversity in a certain region [2].

However, since many organisms respond to environmental characteristics and their changes, it is clear that the number of bioindicator candidates is potentially extremely high—thousands, at least—which complicates their selection. Choosing the “right” bioindicator is by no means trivial because its use will affect policy and management decisions, and undoubtedly, using too many bioindicators may generate contrasting results and be confusing (e.g., [3]). What bioindicator should be used is a sensitive matter that too often leads to partisan arguments, as well as to attempts to “sell” a given organism as a bioindicator because raising its public image in terms of “usefulness” would support its protection. Although this is understandable, it is also unethical because of the decision-making implications of choosing a given bioindicator.

To help prevent such problems, well-established criteria have been formulated, and several proposals exist or have been adopted by the scientific community. For instance, [4] identified a seven-step path that is still valid. Perhaps the most important aspect is that, as established in step 1, the user needs should be determined before developing a list of candidate indicators. It is, then, important to define screening criteria against which indicators should be scored, summarise the results of the scoring process, decide how many indicators are needed and, on such bases, make a final selection [4].

With 1440 species currently known to science, bats are the second most diverse order of mammals and provide a substantial contribution to global vertebrate diversity (Mammal Diversity Database, [5]). There is mounting evidence that bats provide crucial ecosystem services, including suppression of agricultural pest arthropods, pollination, and seed dispersal of an impressive number of plant species, among which several are of high economic value [6,7]. Being sensitive to human action, many bat populations are imperilled; hence, they are legally protected in many regions of the world [8].

Discussion about the use of bats as bioindicators is at least over two decades old. In a 2009 seminal paper, five international bat specialists presented well-grounded arguments on why bats are potentially valuable bioindicators [9]. At least for European bat specialists and conservationists, another two landmarks are represented by the International Symposium on the importance of bats as bioindicators, held in Granollers (Barcelona) in 2012 [10], and a special issue of the journal “Mammalian Biology” devoted to the topic [11]. From the policy viewpoint, EUROBATS (The Agreement on the Conservation of Populations of European Bats, currently binding 37 States Parties on bat conservation) adopted Resolution 6.13, titled “Bats as Indicators for Biodiversity” (https://www.eurobats.org/official_documents/meeting_of_parties/resolutions, consulted on 5 May 2021). In a nutshell, this resolution urges parties to develop national, regional, and pan-European bat biodiversity indicators, facilitate the incorporation of bat data within multi-taxa indicators, support the objective of gathering the data for these indicators and forge cooperation platforms to facilitate data exchange.

In this article, we present the state-of-art of using bats as bioindicators, showing that only a limited number of studies addressed the topic comprehensively and that bats appear to be promising bioindicators, at least in certain environmental contexts. We also highlight limitations and advantages and identify future research and applications, with a special focus on temperate regions.

## 2. Testing Bats as Bioindicators: Where Are We Now?

Despite the considerable attention received by the topic, relatively few attempts have been made to test real-world bioindication applications, and much of the debate on bat sensitivity to environmental changes relies on conservation biology studies showing how bats react adversely to anthropogenic stressors. Few examples of bat-based bioindication schemes are available. In the UK, a “bat index” is part of a suite of organisms used to estimate temporal trends of biodiversity across the country [12]. The index comprises ten bat species trends (two of which are combined), and its recent increase was taken as evidence for a recovery of some bat species after considerable 20th-century population declines [12]. Although this index, along with the others used in the assessment, is highly important to provide a picture of UK biodiversity and its trends (and supposedly indicate “sustainable development”), it does not make it possible to identify the environmental changes that produce the recorded index variation, nor may bats alone summarise the biota responses to human-induced stressors. Hence, in this case, too—one of the few real-world examples of bat bioindication—the use of bats as ecological, or environmental bioindicators appears to be quite limited.

Likewise, an ambitious project that has so far involved nine European countries aimed at developing an index that assesses bat conservation status in the European continent [13]. In that case, a prototype indicator was built from national trends of 16 bat species from nine countries for which hibernacula counts were systematically available. Data were summarised as regional trends and indices, which led to identifying species trends and indices for Europe eventually summarised as a European indicator. As in the UK case, this is a remarkable attempt to reach a good understanding of bat trends in the continent which may inform bat conservation and management. However, the very causes of such trends may be hypothesised but are extremely difficult to be objectively identified: they can be many and waive a tapestry of complex interactions with bat populations, ultimately resulting in the observed responses.

Despite the interest of policymakers and conservationists in employing bats as bioindicators, research lags, and relatively few cases of explicit testing of bat indication performances are available. For example, a simple Scopus search (on 31 January 2021) made using “bats” and “bioindicators” as keywords retrieved 103 documents. Of these, 42 documents addressed the topic in some way, while others were either not strictly relevant or regarded monitoring techniques. In some cases, claiming that bats are useful bioindicators appears more an attempt to emphasise their societal and ecological values and promote conservation than a sound scientific statement based on well-grounded evidence.

Below, we discuss environmental contexts, processes, and management practices for which bats may likely be used as effective bioindicators based on current knowledge.

### 2.1. River Quality

Many studies showed that rivers often provide rich bat species assemblages with key foraging opportunities and that both water quality and riparian vegetation affect food availability [14]. Biological assessment of river quality is among the main bioindication goals worldwide, and it is therefore somewhat surprising that even this highly promising bat bioindication opportunity has not been tested thoroughly. The few studies published showed contrasting results, yet at least some of them suggest that bats might be useful bioindicators of river quality. In a paper [15] set in England and Wales, the authors developed a predictive model of Daubenton’s bat (*Myotis daubentonii*) distribution and abundance at waterway sites based on available monitoring acoustic data. This species is strictly associated with riparian habitats, being perhaps one of the most habitat-specialised bat species in Europe. The model included terms such as biological water quality, waterway width, mean annual discharge, and the presence of trees [15]. The activity was predicted to be higher on larger waterways with abundant woodland nearby but a high amount of site-specific variation was found. The activity was also related to aquatic macroinvertebrate diversity (associated with good chemical water quality). Overall, *M. daubentonii* activity could be predicted from habitat and water quality data, but precision was somewhat too low to apply the model routinely for river quality evaluation. The authors of [16] worked on the same species, this time in the Iberian Peninsula, where they explored correlations between bat activity, as established from monitoring data that had been summarised in the “QuiroRius” index, and two common indices used to characterise river quality (macrobenthos, IBMWP; vegetation, QBR). The weak correlations found showed little agreement among indices, which questions the use of the QuiroRius index for bioindication. This was instead seen as a complementary index that cannot be used alone [16].

Another study [17], carried out in North Carolina, showed more promising results when several bat species were taken into account based on acoustic surveys. Using state-wide water quality information from official biological assessment surveys and urban land cover data, [17] found that bats responded to water quality and urbanisation independently, that responses were species-specific, and that those recorded at the local scale were evident at a landscape scale. The authors concluded that water quality may be used as a predictor for the presence of species of conservation concern, but the study also shows some potential for bat-based bioindication of water quality independent of other landscape stressors such as urbanisation.

**Figure 1:**
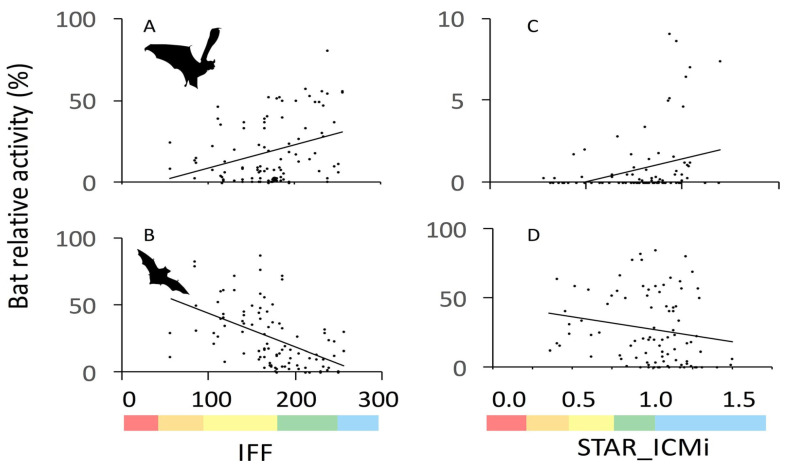
In a study carried out in Italy, bat communities showed significant responses to river quality. The figure shows the relationship between % activity for groups of bat species and the two bioindication indices. (**C**) is a group characterised by similar echolocation calls made of two genera (*Nyctalus* and *Eptesicus*) which were not separated for the purpose of the study; (**A**), (**B**), and (**D**) were groups made by associating species that showed similar responses (“shopping basket” approach) to improve bioindication performances. (**A**) = *Pipistrellus pipistrellus* + *Myotis emarginatus* + *M. nattereri* + *Nyctalus/Eptesicus serotinus* + *Barbastella barbastellus*; (**B**) = *Miniopterus schreibersii/Pipistrellus pygmaeus* + *P. kuhlii*; (**C**) = *Nyctalus/Eptesicus serotinus*; and (**D**) = *M. schreibersii/P. pygmaeus* + *M. daubentonii/capaccinii*. The Fluvial Functionality Index (IFF) is shown on the left, while the macroinvertebrate-based STAR_ICMi index is on the right. River-quality ranges (increasing from left to right) associated to index values are shown below the plots as follows: red = bad, orange = poor, yellow = moderate, green = good, blue = excellent. Reprinted from [18]. Copyright (2018), with permission from Elsevier.

Finally, [18] showed that assemblages of foraging bats along Italian rivers were associated with environmental status and quality (Figure 1). 

River quality was established according to two officially adopted indices, one (STAR_ICMi) based on macrobenthic invertebrate assemblages and another, the fluvial functionality index (IFF), including a suite of biotic and abiotic features to capture river ecosystem health.

In that study too, bat activity was measured acoustically. Higher activity levels were recorded for some species as the values of quality indices increased, while the activity of other species declined; thus, [18] opportunistically pooled together species showing the same trends, which strengthened the indication performances. These results are encouraging; however, as for the others mentioned above, locally adapted bat populations might show different responses in other geographic regions, so the findings of this study warrant confirmation over a broader geographic range.

### 2.2. Farming Practices

Many bat species occur in farmland, and despite the well-known influence of farming practices on bat richness and activity suggesting that these mammals might act as suitable bioindicators in agroecosystems (see [19,20] for a review), few studies have explored this issue. Overall, bats show contrasting reactions to low-intensity vs. conventional farmland, probably because the former management category provides resources and conditions that are more generally favourable to a broad range of wildlife [19,20]. This shows, at least, that bats have the potential to be employed as both ecological and environmental indicators of farming practices. One of the big issues to cope with, however, is that bat responses may potentially be due to many factors, often involving different spatial and temporal scales, and are likely influenced by landscape effects. Difficulties in controlling factors acting “beyond plot” as well as “within-plot” variation (hedgerow network size, the spread of pesticides, water availability, cultivation types, etc.) may help explain the contrasting results obtained when comparing bat activity or species richness between organic vs. conventional cultivations. For instance, out of eight studies specifically designed to pursue this goal, four found more bat species or higher activity at organic sites [21,22,23,24], one detected a few bat species that were more abundant at organic sites, and three found no difference between organic and conventional farmland [25,26,27]. However, after rigorously controlling for the characteristics of landscapes surrounding their study sites, [24] found that organic soybean cultivations had higher bat and insect abundance than conventional ones. Noticeably, insect abundance and dry weight (i.e., food availability) only partly explained differences in bat activity, probably because other factors such as the use of pesticides at conventional sites may have had a substantial influence [24].

### 2.3. Forest Structure and Management

Bats are mostly forest mammals. Many species roost and/or forage in the forest, or use forest patches and corridors for commuting and migration stopovers. The presence and activity of bats in the forest are highly influenced by the forest age and structure, and, in turn, forest management. For example, the availability of suitable tree roosts (and cavities) is highly affected by forest management, which also influences forest heterogeneity, such as the presence of edges, clearings, and undergrowth—features providing foraging opportunities to different bat species, depending on their hunting strategies [28]. For example, coppice (in which trees are cut to ground level and regrow agamically) is a form of management that implies high tree density and small tree diameters, making the forest unsuitable for both roosting and foraging at its core. However, it also generates clearings and edges, which favours feeding by edge specialists such as, in Europe, pipistrelle bats [29]. Well-preserved high forest, hosting many veteran or dead trees rich in cavities and enough space for core foraging, is exploited by many tree-roosting species and ground or foliage gleaners [29]. Therefore, it is expected that bats will exhibit species-specific responses to forest management and that this will concern both roosting and foraging behaviour, setting the scene for the use of these mammals as indicators of forest management. Despite the overwhelming literature that is available on the ecology of forest bats and their conservation implications (e.g., [30]), explicit attempts to use bats as bioindicators in this context are surprisingly rare. In one of such cases, not only were bats found to respond clearly to different management options applied to a range of forest types and structures, but such effects were detected on total bat activity expressed as the sum of all bat passes recorded [31]. This offers a robust, taxonomy-independent approach to bioindication in forest ecosystems. There is also mounting evidence that bats exhibit responses to wildfires [32,33,34], so bats may offer considerable potential for bioindication of after-fire recovery patterns.

### 2.4. Urbanisation

Urban areas filter out many mammal species, including bats [35]. Notwithstanding this impact, bats still represent the most numerous urban-dwelling order of mammals, with ca. 80 species out of the over 1400 currently described [35]. At the landscape level, there is a clear negative relationship between the amount of urban space in the landscape and bat richness, and bat richness typically declines along gradients of increasing urbanisation. Although urban areas provide several bat species with considerable roosting opportunities in buildings, prey is often scarce—it is mostly concentrated in green or blue spaces (e.g., [36])—and artificial illumination repels most bat species, apart from a handful of species that exploit insects attracted at lights [37]. Moreover, anthropogenic noise [38] and predation by opportunistic animals such as domestic cats also have adverse effects on bats. As for farmland, singling out the specific drivers that elicit responses by bats can be tricky, but also in urban habitat, there is great potential for bat bioindication, and species richness or community composition are likely to prove effective, at least to highlight urbanisation gradients or urban sustainability.

### 2.5. Bioaccumulation

Bats typically accumulate chemicals such as pesticides and heavy metals from their food, which is a threat to bat survival but also offers an important opportunity for bioindication [39,40]. For example, flying foxes in Australia proved to be excellent bioindicators of environmental metal exposure: kidney and fur lead concentrations in recent specimens were lower than those recorded in samples taken in the early 1990s. Likewise, insectivorous bats in China were used to study mercury bioaccumulation [41]. In aquatic ecosystems, contaminants are transferred from freshwater sediments to bats through their insect prey, and a water habitat specialist such as *M. daubentonii* provides compelling evidence about bat bioindication potential in this context. Fur samples from this species were taken before and after remediation work (sediment dredging) was carried out at a German pond [42]. Measures made on the sediment after remediation showed only a weak decline in heavy metal content, while a pronounced decline was recorded from bat fur analysis, confirming that this bat species is an effective bioindicator of metal contamination in aquatic ecosystems [42]. The possibility of carrying out analysis on fur makes the approach non-invasive, thus being applicable to mammals at risk, such as bats.

### 2.6. Climate Change

Bats are widespread across the globe and their physiological requirements are greatly influenced by ambient temperature, which affects life cycle characteristics such as hibernation and reproduction [43]. Bats are therefore likely to react to climate change, yet responses have so far been recorded only in a few species (e.g., [44]). The bat’s large body surface makes dehydration a considerable risk that must be prevented by drinking nightly, so climate change-driven disappearance of drinking sites poses a further threat to bats. This dependence upon climate makes bats excellent candidate organisms to indicate biotic responses to climate change, as proposed by [45]. This idea is pursued by the CA18107 COST Action “ClimBats” (www.climbats.eu, accessed on 10 May 2021), a European-Union funded action that, among its several goals, aims at designing monitoring networks spread across Europe to monitor compositional changes in bat assemblages such as to reflect responses to climate change.

### 2.7. Surrogate Taxa

Surrogate taxa (or biodiversity indicators) are groups of organisms whose species richness may effectively indicate the species richness of other taxa [46] providing a cost-effective way of capturing overall diversity and informing conservation planning, such as in establishing priorities for the selection of areas to be protected. Several groups may also be pooled together to strengthen their bioindication power, in a so-called “shopping basket” approach [47]. It is a common (anecdotal) experience that greater numbers of bat species are found in well-preserved sites where the overall biodiversity of animal communities is also high, whereas degraded environments tend to host a much lower number of bat species [48]. On such bases, using bats as a surrogate taxon would be tantalising; however, while few studies have tested their performances in this respect, such studies also provided contrasting results.

Perhaps the most promising results regard a study carried out in Denmark which used a comprehensive (434) species dataset available from atlases of bats, butterflies, birds, amphibians, reptiles, large moths, and click beetles [49]. In that study, bats and large moths proved as the most robust taxa in selecting grid cells that included the greatest richness of other taxa. In French forests, however, although bats were the most congruent taxon for alpha-diversity with bryophytes and ground beetles, they were classified as a low-cost yet inefficient surrogate group [50]. Likewise, [51] examined four tropical forest types in the Philippines and restricted their analysis to bats, birds, and trees, showing results that discourage the use of bats as a surrogate taxon in those contexts at moderate (100 × 35 km) spatial scales. Similarly, in Amazonian tropical forests, bats did not show good performances as biodiversity indicators, possibly—as the authors put it—because of bats’ specific responses to land-use change and high mobility. In Germany, bats also showed limited potential to predict hotspots of phylogenetic (rather than taxonomic) diversity across species groups including dragonflies, grasshoppers, butterflies, and birds [52]. The study advised against using one taxon as a surrogate for others and highlighted that phylogenetic diversity correlated negatively with the amount of broadleaf forest, probably because specialised forest bats are closely related (such as those in the genus *Myotis*).

All the above-mentioned studies considered different geographic regions, ecosystem types, spatial scales, taxonomic assemblages, data sources or sampling methods, and sometimes even tackled varying goals, which may explain why they led to contrasting findings and, overall, provided a confused general picture.

## 3. Potential Limitations to the Use of Bats as Bioindicators

**Figure 2:**
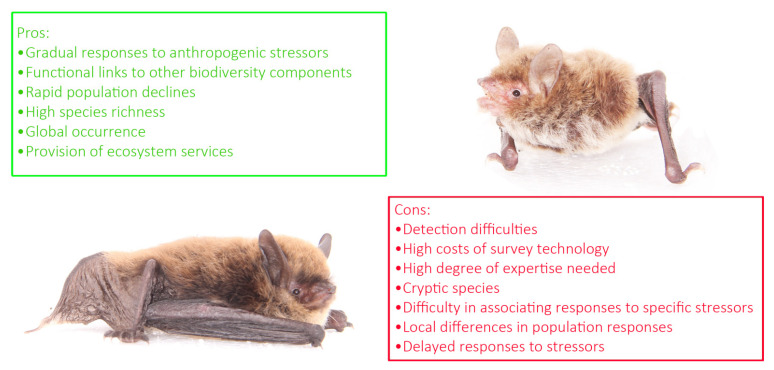
A schematic list of pros and cons of using bats as bioindicators.

The use of bats as bioindicators has pros and cons (Figure 2). The authors of [5] identified eight points on which bases bats would represent excellent bioindicators.

These comprise: (a) relative taxonomic stability; (b) wide geographic ranges; (c) rich trophic diversity; (d) provision of key ecosystem services, (e) graded responses to environmental alteration correlated with those of other biodiversity components, such as insects; (f) rapid population declines due to slow population growth; (g) possibility of measuring several variables (population size, feeding activity, etc.); and (h) the role of bats as reservoirs of emerging infectious diseases whose epidemiology could reflect environmental stress.

Such criteria are therefore a mix of intrinsic features of bat natural history, their correlation with anthropogenic stressors, and practical sampling aspects. While this analysis provides a general picture of the potential value of bats as bioindicators, some of these aspects, as well others, merit further discussion, especially in light of new knowledge on bat biology, ecology, and technology applied to sampling.

### 3.1. Taxonomical Issues

Bats show relative taxonomic stability compared with many other animal taxa; however, in the last years, their taxonomy has proven relatively fluid due to the advent of molecular approaches that have resolved phylogenetic relationships and revealed many cryptic species (e.g., [53,54]). Even if we limit our analysis to the European region alone, many new cryptic species appeared on the scene in the last few decades. One of the most striking examples is offered by an abundant and widespread European species, the former “common pipistrelle” *Pipistrellus pipistrellus*, which in 1997 was split into two valid species (also differing in echolocation, behaviour, and ecology yet highly similar in morphology), *P. pipistrellus stricto sensu* and *P. pygmaeus* [55]. Since then, molecular analysis revealed the existence of several other cryptic species in Europe, including new *Plecotus* and *Myotis* species [56]. The difficulty of telling apart cryptic species in the field may hinder their usefulness as bioindicators. For example, in the UK biodiversity index, the two cryptic *Myotis* species *M. mystacinus* and *M. brandtii* are lumped together into a single taxon trend due to the difficulty of field distinction between them [12]. Of course, this tendency may not be representative of species-specific population trends because a population decline of one species might be masked by an increase of the other. Moreover, a general consideration for cryptic species pairs or groups is that despite their high morphological similarity, they often show marked ecological differences [57], which leads to different responses to environmental stressors. Using such bat species for bioindication would therefore require reliable identification, a task that may be too specialised, time-consuming, or expensive for large-scale sampling or monitoring.

### 3.2. Sampling Limitations

Several variables may indeed be measured from bats, which increases their usefulness as bioindicators. However, from a technical viewpoint, bats are not easy to sample. They may be observed directly in their roosts or captured in foraging or drinking sites, as well as along commuting routes, but they are nocturnal, elusive, often evade capture, and are also highly sensitive to disturbance [29]. For this reason, in most countries, permits are needed for bat capture and handling. Capture is necessary to obtain the bat’s DNA, normally from skin tissue sampling, and identify confidently cryptic species (e.g., [58,59]. Bat capture and field identification require well-trained personnel, which limits the involvement of volunteers, rangers, and other non-specialised staff. A widespread alternative to capture is acoustic surveying and monitoring, in which echolocation (and, sometimes, social calls) are recorded for subsequent species identification [60]. Variables such as feeding rates may be inferred from counting “feeding buzzes”, sequences of echolocation calls broadcast by bats on prey approach [61]. Bat echolocation calls, however, show high intraspecific and even intraindividual variation, while call design converges among species in response to overlapping sensory and ecological requirements [62]. This is the reason why not all species can be recognised with confidence, and despite the advent of automatic classification, it is still wise to refrain from identifying all calls to species. From the bioindication viewpoint, species misclassification is a serious concern, and lumping together similar echolocation calls that cannot be classified to the species level might not provide meaningful information [63]. In other words, acoustic identification may not always provide taxonomic sufficiency, i.e., the degree of identification requested to detect differences in community composition or relative activity that characterise different environmental conditions [64]. Acoustic surveys require the employment of ultrasound detectors and recorders, as well as specialised software for sound analysis, whose costs are still not negligible.

### 3.3. Disentangling Cause-Effect Relationships

**Figure 3:**
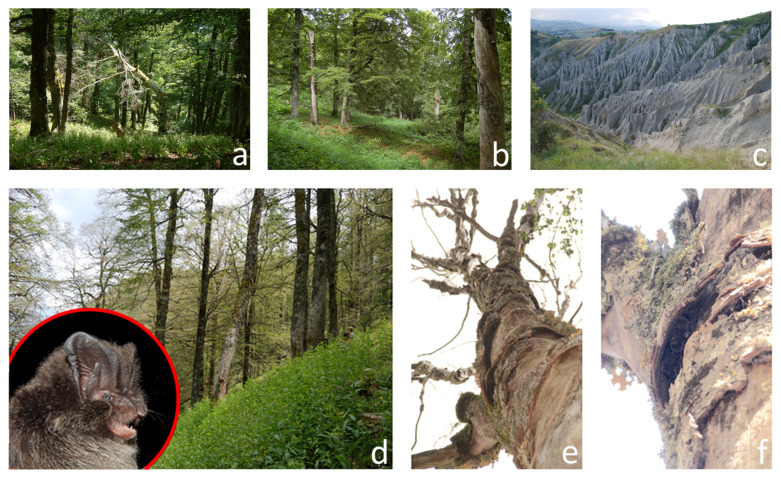
Barbastelle bats (*Barbastella barbastellus*), often deemed forest bats typically associated with unmanaged forest (**a**), may in fact occur in logged sites (**b**) or even in clay badlands (**c**) characterised by few or no trees. The species is sensitive to multiple spatial scales, from landscape/habitat (**d**) to tree (**e**) and cavity (**f**) types. In (**f**), a small reproductive group can be noticed roosting underneath the flaking bark of a beech snag.

A highly desirable (yet not always necessary) property of a bioindicator is a narrow ecological niche so that the organism will be present (or absent) only under certain environmental conditions, providing discrete responses. For example, among aquatic arthropods, Ephemeroptera, Plecoptera, and Trichoptera (EPT) are highly intolerant to water pollution, so in many cases, their presence can be used to characterise high water quality, and they tend to be absent in polluted rivers [65], albeit with some exceptions (e.g., [66]). As for bats, this is, in many cases, not possible. First, relying on species presence may lead to errors due to limited detectability: bats evading capture or weak echolocators overlooked in acoustic surveys may lead to false absences [67]. Second, especially when using acoustic data, the resulting often-coarse taxonomic resolution may hinder species-specific associations to certain habitats, environmental conditions, or landscape management practices. Even more importantly, most bat species are multiple-habitat specialists [68] that occur in a relatively broad spectrum of environmental conditions. This holds even for relatively specialised bat species, let alone generalists such as, e.g., pipistrelles [69]. For example, the barbastelle bat (*Barbastella barbastellus*), often regarded as a forest specialist, finds optimal reproductive habitat in the unmanaged high forest, so one might be tempted to use barbastelle presence to indicate relatively undisturbed forest (e.g., [70,71]). However, barbastelle bats also frequently occur in logged forests and may even be found on rocky islands with little woody vegetation [72] or in forestless clay badlands ([73]; Figure 3). The species shows *continuous* rather than *discrete* responses to forest structure and management, such as changes in the sex ratio or density of reproductive groups [74].

Although such continuous responses may still be employed for bioindication, value ranges should be defined, and categorisation might be arbitrary and unlikely to remain valid across broad geographic regions or environmental gradients. Variables such as species richness, however, can be more easily used to categorise responses to habitats, land-use change, or environmental stressors, such as urbanisation gradients [75]. Moreover, bats fly over long distances, so they may often cross unsuitable habitats to reach their roosting or foraging sites—one more reason why detecting a given bat species in a certain habitat or under a specific environmental condition might tell us little.

Bats find their resources in a range of habitats: roosts, food, and water may occur in separate habitats, so bats may show responses to alteration of any such habitats. Responses to habitat changes may occur even if alteration concerns non-bat habitat. Bat commuting routes, such as those connecting roosts with foraging sites, often cross habitat that contains no bat resource but is still vital to bats to support their movement. Human action may sever commuting routes, for instance, through light pollution [37], and this may have adverse effects on the bat energy budget and ultimately fitness, even if the affected habitat does not contain any resource. Insectivorous bats normally only feed on adult insects, whose abundance will depend on prey reproductive success and larval survival [76]. Therefore, if larval and imaginal stages of prey occur in a different habitat, bats might be affected by the alteration of habitat they do not use directly, provided the latter is important for prey reproduction [77]. Furthermore, bats are sensitive to multiple spatial scales. Following with the example of the above-mentioned barbastelle bat, its habitat requirements span over a broad spatial range (Figure 3), from large-scale connectivity (forest corridors connecting the main roosting areas and mountain ridges delimiting the latter) to specific features of the habitat around the roost tree, the tree itself and the roost cavity [70,71,78,79,80].

However, why is all this a problem for bioindication? Effective bioindication requires clear reactions to well-identified causes, i.e., a clear picture of which environmental factor or stressor will cause an observed response. Therefore, despite bats’ high sensitivity to changes in environmental factors, disentangling the rich tapestry of stressors that often act synergically on bat activity and/or population size is a difficult exercise. Although statistical models such as GLMMs may cope with multiple-variable systems, for instance taking into account the role of several spatial scales (e.g., [81]), the results are often not sufficiently straightforward to translate into a practical bioindication approach. Bat activity is also highly influenced by factors such as temperature [82], wind speed [83], and, according to some authors [84], even moon phase, so that it may vary substantially even between consecutive days or within hours. On a cold spell, bats may even not show up, resulting in false absence. Models testing activity responses need to accurately include such factors.

### 3.4. Responses May Be Influenced by Local Adaptations

Finally, bat species often cover wide geographic ranges, which is, in principle, a desirable bioindicator quality [5]. However, this does not mean that intraspecific responses to environmental stressors will stay equal across a species’ whole distribution. For example, intraspecific differences in bat climatic tolerance may be due to local adaptation and result in different population-scale responses [85]. Extrapolating the interpretation of observed responses (and bioindication applications) to different areas within a species’ geographic range may therefore be misleading, which prevents geographic generalisation of bat bioindication approaches.

### 3.5. Delayed Responses to Environmental Stressors?

Ideal bioindicators should exhibit prompt responses to environmental conditions and their changes, which allow decision-makers to take action swiftly to reverse the trend if needed. From this viewpoint, bats are promising since their low reproduction rates make demographic recovery slow and population declines conspicuous (e.g., [86]). However, one study carried out in Hokkaido (Japan) showed that current bat activity was influenced by past landscapes, i.e., bats adapted to hunt in open space (a “25 kHz” phonic group) were more active where the broadleaved forest was scarcer in the 1950s, and open spaces used to be dominant [87]. This would represent a legacy of once optimal (open-space) sites to which bats remained faithful despite subsequent habitat transformation. On such bases, [87] suggested that bats may undergo time-lagged effects of past environmental conditions, but the study did not control for other factors that might potentially affect the bat population size and activity.

## 4. Conclusions

There is an ever-growing number of biologists and ecologists who are uncomfortable with proposals of new organisms as bioindicators because sometimes the proposers seem to oversell a certain bioindicator to raise its public profile and achieve better protection rather than to respond to real indication needs. This is risky because it weakens the usefulness of bioindicators and their credibility. Therefore, we should first establish what questions bats can answer, or in which habitats they may act as bioindicators. For example, based on what we discussed, bats are promising bioindicators in riverine systems, and their practically constant presence along rivers, where they act as top predators, fully highlights this potential. The bat’s high position in the trophic chain also makes bats especially useful to monitor the presence of contaminants such as pesticides or heavy metals. Bats are protected, so killing them for science poses conservation and ethical problems and is illegal in many countries [88]; however, as we discussed, the presence and concentration of heavy metals may be assessed humanely. Moreover, using bats that die in rehabilitation centres or are killed by, e.g., windfarms provide useful material to carry out measurements of heavy metals or pesticides in bat organs [88].

Bats also offer promising responses to forestry or farming practices, and the main problem of disentangling the specific management factors that cause responses may likely be overcome through ad hoc research [9]. The high sensitivity of bats to temperature changes and water availability also makes bats potentially excellent indicators of climate change. In our view, however, identifying habitats and processes where bats may do well, or better than other organisms as indicators needs new research perspectives, shifting from conservation biology studies to specifically designed protocols that may assess bat responses and standardise their measurement and interpretation. Moreover, networking among researchers is crucial in covering large geographic regions and accounts for location-biased responses that might hinder general patterns. The academic pressure for “novelty” in scientific articles may seriously discourage researchers from replicating studies carried out elsewhere in the geographic regions where they work, but this is an obstacle that the research environment needs to eliminate to test the bioindicator properties of bats. Standardising methods will be another key step towards using bats as bioindicators, at least within indicator types or habitats, perhaps appreciating that bats might in some cases be useful indicators on a small but not large geographic scope.

Other problems that hinder a standardised use of bats as bioindicators are related to the costs of the technology involved in (especially acoustic) bat surveys and the often-coarse taxonomic resolution provided by echolocation call analysis. The former problem is likely to be greatly mitigated, if not solved soon, by the advances in survey technology [89,90]. Taxonomic insufficiency, however, will be overcome only by developing robust indicators, for instance, by testing responses by broader “phonic types” (groups of species sharing similar calls) or overall bat activity: this would circumvent all taxonomic issues. Otherwise, indication applications should be restricted only to species that are identified with confidence.

As many bat species are at risk, their systematic monitoring is mandatory in many countries to assess population trends, and in the EU, bat monitoring is an obligation arising from Article 11 of 92/43/EEC “Habitats” Directive. Therefore, developing monitoring methods that may both estimate bat conservation status and provide applications to bioindication would be a cost-effective approach to accomplish two objectives with one action.

## Data Availability

Not applicable.
